# A Rare Case of Porocarcinoma and Trichoblastoma Arising in a Nevus Sebaceus of Jadassohn

**DOI:** 10.1155/2021/7598086

**Published:** 2021-03-03

**Authors:** Drew C. Mitchell, Gina J. Kuehn, Glynis A. Scott, Timothy D. Doerr, Francisco Tausk

**Affiliations:** ^1^University of Rochester School of Medicine & Dentistry, 601 Elmwood Avenue Box 400, Rochester, NY 14642, USA; ^2^Department of Dermatology, University of Rochester Medical Center, 40 Celebration Drive, Rochester, NY 14642, USA; ^3^Department of Pathology, University of Rochester Medical Center, 601 Elmwood Avenue Box 626, Rochester, NY 14642, USA; ^4^Department of Otolaryngology, University of Rochester Medical Center, 601 Elmwood Avenue Box 629, Rochester, NY 14642, USA

## Abstract

Nevus sebaceus of Jadassohn, or “organoid nevus,” is a common, benign hamartoma of the skin consisting of epithelial and adnexal components. Its natural history and association with neoplastic growths is well documented. The majority of concomitant neoplasms are benign—trichoblastoma and syringocystadenoma papilliferum are most frequently discovered—but malignant tumors have been described. We present the case of a 58-year-old male with a congenital nevus sebaceus of Jadassohn on his left parietal scalp that had been enlarging, changing color, and bleeding over the prior year. Clinical exam and histology disclosed the presence of a trichoblastoma and porocarcinoma arising within the nevus sebaceus. Porocarcinoma is a rare, intermediately aggressive, malignant eccrine gland tumor that is frequently metastasized at presentation. Otolaryngology performed wide local resection with sentinel lymph node biopsy. This case highlights the diversity of tumors associated with nevus sebaceus of Jadassohn, potential for malignant expansion, and necessity for close monitoring and maintaining a low threshold for biopsy in evolving lesions.

## 1. Introduction

Nevus sebaceus of Jadassohn (NSJ), also known as “organoid nevus,” is a common, benign hamartoma composed of abnormally developed hair follicles, papillomatous epidermal hyperplasia, and ectopic apocrine glands that presents at birth or in early childhood [1–4]. Typically, NSJ presents as a single, sharply demarcated yellow-orange plaque on the scalp, often with accompanying alopecia [3, 4]. It is identified in approximately 0.3% of neonates [4] and has an incidence of 0.05–1.0% in dermatology patients [2, 4].

The natural history of NSJ and its association with neoplastic growths is richly documented such that ‘Tumoral' is the accepted final developmental stage [4]. Although the overwhelming majority of neoplasms that arise in NSJ are benign, malignancies have been consistently discovered, and the risk of neoplasia—benign or malignant—increases with age [1–3]; however, to the best of the authors' knowledge, this is the first multitumoral case involving a porocarcinoma. We report a rare case of porocarcinoma and trichoblastoma arising in an NSJ.

## 2. Case Report

**Figure 1:**
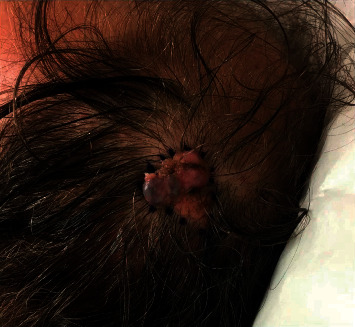
Clinical appearance of left parietal lesion at presentation to dermatology clinic.

A 58-year-old male presented for an evolving mass on his left scalp. The mass was noted at birth and had been enlarging, changing color, and bleeding over the past 12 months. On exam, a 3.0 cm × 3.0 cm yellow-to-tan verrucous, papulated plaque with central, large pink, and violaceous-to-blue nodules was present on the left posterior parietal scalp with overlying alopecia **(**Figure 1**)**.

**Figure 2:**
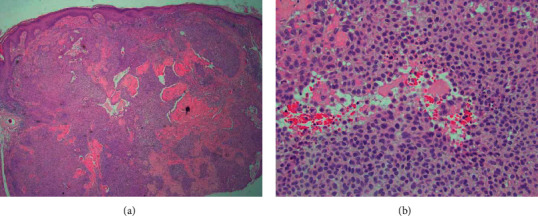
(a) Scanning micrograph of the porocarcinoma shows an expansile tumor arising from the epidermis with extension deep into the reticular dermis (hematoxylin and eosin, 4x). (b) Higher power shows focal necrosis of the tumor which displays cytologic atypia, including mitotic figures (hematoxylin and eosin, 20x).

A shave biopsy of the entire lesion was performed. Pathology disclosed changes consistent with an organoid nevus with two distinct neoplasms within. A basaloid tumor arising from the epidermis with a focal ribbon and pigmented pattern consistent with trichoblastoma and an adjacent downgrowth of atypical squamous epithelium with dysplasia and easily identified mitotic figures with a glassy stroma and eccrine differentiation consistent with porocarcinoma (Figures 2(a) and 2(b)). Immunohistochemical stains against cytokeratin 7 highlighted portions of the tumor.

The patient was referred to otolaryngology for wide local resection with sentinel lymph node biopsy. There was no parotid gland enlargement or palpable anterior or posterior cervical lymphadenopathy. Wide local excision (6 cm × 6 cm with 1.5 cm to 2 cm margins) was performed with sentinel lymph node biopsy from the left tail of the parotid identified through nuclear medicine. Reconstruction was with opposing transposition flaps.

Surgical pathology revealed no residual porocarcinoma in the scalp specimen and negative sentinel lymph nodes; therefore, no further treatment, including chemotherapy, was indicated. Three years later, the patient remained disease free.

## 3. Discussion

Nevus sebaceus of Jadassohn is a common, congenital lesion that overwhelmingly presents on the head with a special affinity for the scalp and persists throughout life [1–3, 5]. Generally, it evolves from a smooth bald patch in infancy to a raised cerebriform lesion in adulthood [4]. While not fully elucidated, the etiology of NSJ is thought to result from stem cell genomic mosaicism that expands the distribution of the lines of Blaschko [3].

NSJ's association with secondary neoplasms is well established. It is estimated that 10–21% of NSJs develop a tumor [1, 2, 5]; however, since there are no prospective follow-up studies of NSJ, it is likely that this number is biased and overinflated. Moreover, greater than 90% of these tumors are benign [1–5].

Historically, it was thought that basal cell carcinomas (BCC) were the most common neoplasm complicating NSJ, but studies from the early 1990s and forward have confirmed that what was previously believed to be BCC were, in fact, misdiagnosed trichoblastomas [1, 5]. It is now accepted that trichoblastoma and syringocystadenoma papilliferum are the two most common neoplasms in NSJ, each occurring in approximately 5% of NSJ [1, 2, 4, 5]. BCC is the most common malignant neoplasm associated with NSJ [2, 5]. NSJs are often surgically removed for cosmesis but due to low rates of tumor development, especially malignant, prophylactic removal is no longer recommended [3, 4].

Porocarcinoma is the most common malignant eccrine gland tumor and accounts for an estimated 0.005–0.01% of all cutaneous tumors [6, 7]. Its etiology is poorly understood, but it can arise primarily or secondarily; preexisting eccrine poroma, exposure to chemical agents, chronic light exposure, and immunosuppression are all thought to be potential contributing factors for its genesis [7, 8].

Porocarcinomas present as a solitary nodule or mass involving the lower extremities—head/neck and upper extremity lesions are seen about equally [6–9]. The scalp is an uncommon site of presentation with less than 20 reported cases in the literature [9]. There is a strong predilection toward older age groups (7^th^-8^th^ decade of life) and inconsistent reports regarding gender preference [7–9].

Porocarcinomas are an intermediately aggressive tumor with metastatic disease at presentation in up to 31% of cases, most frequently involving regional lymph nodes (60%) and the lungs (13%) [6–8]. When metastases are discovered, mortality is high: 80% in distant disease and 65–67% when lymph nodes are involved [6, 7]. Wide local resection is the mainstay of treatment with adjuvant chemoradiotherapy added for metastatic disease or local recurrence [6–9]. Despite therapy, there is a 20% recurrence rate [7, 9].

The only case describing a porocarcinoma developing in an NSJ was reported in 1985 by Tarkhan and Domingo where the lesion also arose on the scalp but had postauricular extension and metastasis [10]. Previous reports of porocarcinomas document that those located on the head/neck had the lowest incidence of lymph node metastasis at the time of diagnosis, as compared with other anatomical locations [7]. It is unclear if porocarcinomas arising within NSJ obey the same tendencies as those developing elsewhere. Additionally, NSJs on the scalp are more regularly complicated by neoplastic growths than NSJs in other locations [5].

In our case, the patient demonstrated a porocarcinoma that arose adjacent to a trichoblastoma. There are several reports of multiple neoplasms developing in a single NSJ [1, 2], and trichoblastoma is the tumor most likely to cooccur with another neoplasm [2]. Although the relationship of trichoblastoma to multiple neoplastic growths may simply be a function of its relatively high incidence in NSJ, this would not explain why it is seen more commonly than in syringocystadenoma papilliferum. To the best of our knowledge, this is the first reported case of the simultaneous appearance of a porocarcinoma and another tumor arising in an NSJ.

This case highlights the potential for malignant expansion in NSJ, the diversity of tumors that arise within it, and the clinical necessity of closely monitoring the evolution of these lesions and maintaining a low threshold for biopsy.

## Data Availability

No data were used to support this study.
